# School-based intervention to enable school children to act as change agents on weight, physical activity and diet of their mothers: a cluster randomized controlled trial

**DOI:** 10.1186/s12966-016-0369-7

**Published:** 2016-04-06

**Authors:** Nalika Gunawardena, Kayo Kurotani, Susantha Indrawansa, Daisuke Nonaka, Tetsuya Mizoue, Diyanath Samarasinghe

**Affiliations:** Department of Community Medicine, University of Colombo, No.25, PO Box. 271, Kynsey Road, Colombo-08, Western Province Sri Lanka; Department of Epidemiology and Prevention, Center for Clinical Sciences, National Center for Global Health and Medicine, 1-21-1, Toyama, Shinjuku-ku, Tokyo Japan; The Foundation for Health Promotion, No.21/1 Kahawita Road, Attidiya, Dehiwala, 10350 Western Province Sri Lanka; Department of Global Health, School of Health Sciences, University of the Ryukyus, 1 Senbaru, Nishihara-cho, Nakagami-gun, Okinawa Japan; Department of Psychological Medicine, University of Colombo, Sri Lanka, No.25, PO Box. 271, Kynsey Road, Colombo-08, Western Province Sri Lanka

**Keywords:** Diet, Physical activity, Mother, Obesity, Randomized controlled trial, Student, Sri Lanka

## Abstract

**Background:**

School health promotion has been shown to improve the lifestyle of students, but it remains unclear whether school-based programs can influence family health. We developed an innovative program that enables school children to act as change agents in promoting healthy lifestyles of their mothers. The objective of this study was to examine the effect of the child-initiated intervention on weight, physical activity and dietary habit of their mothers.

**Methods:**

A 12-month cluster randomized trial was conducted, with school as a cluster. Participants were mothers with grade 8 students, aged around 13 years, of 20 schools in Homagama, Sri Lanka. Students of the intervention group were trained by facilitators to acquire the ability to assess noncommunicable disease risk factors in their homes and take action to address them, whereas those of the comparison group received no intervention. Body weight, step count and lifestyle of their mothers were assessed at baseline and post-intervention. Multi-level multivariable linear regression and logistic regression were used to assess the effects of intervention on continuous and binary outcomes, respectively.

**Results:**

Of 308 study participants, 261 completed the final assessment at 12 month. There was a significantly greater decrease of weight and increase of physical activity in the intervention group. The mean (95 % confidence interval) difference comparing the intervention group with the control group was −2.49 (−3.38 to −1.60) kg for weight and −0.99 (−1.40 to −0.58) kg/m^2^ for body mass index. The intervention group had a 3.25 (95 % confidence interval 1.87–5.62) times higher odds of engaging in adequate physical activity than the control group, and the former showed a greater number of steps than the latter after intervention. The intervention group showed a greater reduction of household purchase of biscuits and ice cream.

**Conclusions:**

A program to motivate students to act as change agents of family’s lifestyle was effective in decreasing weight and increasing physical activity of their mothers.

**Trial registration:**

Sri Lanka Clinical Trials Registry SLCTR/2013/011.

**Electronic supplementary material:**

The online version of this article (doi:10.1186/s12966-016-0369-7) contains supplementary material, which is available to authorized users.

## Background

Noncommunicable diseases (NCDs) are major causes of death globally, accounting for 68 % of total deaths worldwide in 2012 [1] and 54 % of disability-adjusted life years worldwide in 2010 [2]. Nearly three quarters of all NCD deaths and 82 % of premature deaths (death below 70 years) occur in low- and middle-income countries, and the greatest increase in the number of NCD deaths between 2000 and 2012 was recorded in the WHO South-East Asia Region, from 6.7 to 8.5 million [1]. In Sri Lanka, 75 % of all deaths were due to NCDs, including cardiovascular disease (40 %) and cancer (10 %); the probability of dying between ages 30 and 70 years from the 4 main NCDs (cardiovascular disease, cancer, diabetes mellitus and chronic respiratory disease) was 18 % [1].

NCDs are largely caused by behavioral risk factors including unhealthy diet, physical inactivity, tobacco use and excessive use of alcohol [3], which are major pervasive problems during economic transition and rapid urbanization. Obesity-a consequence of an imbalance between energy intake and expenditure- increases risk of death from major NCDs including cardiovascular disease and cancer [4, 5]. Therefore, the identification and control of these modifiable risk factors are fundamental for the prevention of NCDs. In Sri Lanka, 19 % of men and 33 % of women were overweight, defined as a body mass index (BMI) of ***≥***25 kg/m^2^, and the prevalence of insufficient physical activity was 17 and 30 % in men and women, respectively [1]. These data indicate that in Sri Lanka, women are more likely to be obese and physically inactive than men, underscoring the need for intervention to improve women’s lifestyle.

School-based intervention has been shown to be effective for children in increasing physical activity [6], decreasing consumption of sugar-sweetened beverages [7], and preventing obesity [8]. Besides, children have potential to act as change agents in promoting cardiovascular health of their parents. This notion has been supported by recent randomized trials, which reported the effect of educational programs for students on the improvement of CVD risk score of their parents [9] and on the reduction of salt intake and blood pressure among adults of their family [10]. In our previous intervention in Sri Lanka, we found that students were remarkably capable of engaging their families in efforts to improve wellbeing ([11], and unpublished observations by Gunawardana N, Indrawansa S, Mizoue T, Nanri A, Nonaka D, Rajapakse L, Samarasinghe D), suggesting a role of children in modifying lifestyle of their family. Given a close mother-child relationship, school-based intervention would be an efficient and valuable approach for promoting health of young mothers. To further explore whether school children can contribute to the improvement of health behaviors and weight reduction of their mothers, we developed an innovative program that enables school children to act as change agents in promoting healthy lifestyles of their mothers. Here, we report the effect of this 12-month intervention on changes of physical activity, dietary habit and weight of mothers.

## Methods

### Study design

This study is a cluster randomized controlled trial among Sri Lankan mothers with a school-aged child, with school as the cluster. The intervention period was 12 months. The study protocol was approved by the Ethics Review Committee of Sri Lanka Medical Association. Written informed consent was obtained from mothers who agreed on the participation of themselves and their children into the study. The study was registered in the Sri Lanka Clinical Trials Registry (SLCTR/2013/011).

### Study setting and participants

Schools in Sri Lanka are classified according to levels of teaching grades and the streams of study. Specifically, Type 1C schools contain classes up to Advanced Level General Certificate of Examination, while Type 2 schools contain classes only up to Ordinary Level General Certificate of Examination. The present study was conducted among Type 1C and 2 schools in Homagama Educational zone. Grade 8 students, aged around 13 years, of participating schools received an educational program at school, whereas their mothers were invited to pre- and post-intervention surveys to assess the effect of intervention. Students were excluded from the intervention if their mother was pregnant or under special diet plans of a dietician or medical professional. The conduct of the study was officially approval by the Provincial Minister of Education and the Provincial Director of Education, who informed the Zonal Education Office in Homagama to work with the study team in the recruitment of schools. None of the schools we approached declined the invitation.

### Sample size

The sample size estimation was based on the objective to compare between the intervention and control groups the proportion of women who would be involved in adequate physical activity at the end of the intervention. A group of mothers with grade 8 students in a school were considered a cluster. The number of clusters required per group was assumed as *c* and the cluster size was decided as 15 based on the expected minimum number of students per class. In the estimation of sample size, we calculated the number of clusters required per group according to the formula for cluster randomized trial [12]. If *n* individuals are sampled in each cluster, *c*, the number of clusters required, is given by

$$ c=1+{\left({z}_{\alpha /2}+{z}_{\beta}\right)}^2\left[{\pi}_0\left(1-{\pi}_0\right)/n+{\pi}_1\left(1-{\pi}_1\right)/n+{k}^2\left({\pi_0}^2+{\pi_1}^2\right)\right]/{\left({\pi}_0-{\pi}_1\right)}^2 $$

where *π*_1_ and *π*_0_ are the true proportions in the presence and absence of the intervention, with *k* being the coefficient of variation of true proportions between clusters within each group.

The proportion of those who engaged in adequate physical activity in the control clusters was estimated as 33.3 %, considering the level of physical activity among Sri Lankan adult females [1] (*π*_0_ =0.33). In the absence of empirical data to estimate *k*, 0.3 adopted as *k* [12] to imply that the true rates in the control clusters would vary roughly between *π*_0_ (1 ± 2 *k*), i.e., 13.2 and 52.8 %. In this study, we assumed that the intervention would increase the proportion of mothers with adequate physical activity by 20 %, estimating the *π*_1_ as 53 % (0.53). Furthermore, the assumption of equal *k* in the two groups implies that cluster rates in the intervention group would vary between 21.2 and 84.8 % with the assumed *k* of 0.3. The estimated value of *z*_*α*/2_ was 1.96 corresponding to the level of significance of *P* = 0.05 and estimated value for *z*_*β*_ was 0.84 corresponding to power of 80 %. With these data, *c* was estimated to be around 11.

### Selection of schools and randomization

There were 95 Type 1C and 2 schools in Homagama Educational zone. For each type of school, we created a list of schools, which were sorted in alphabetical order and assigned consecutive numbers. We then randomly selected 20 schools (4 and 16 from Type 1C and type 2 schools, respectively). If a school selected was located within 10 km of any school which was already selected (regardless of school type), we replaced the school by another. Within the same school type, we randomly assigned half of the schools to intervention and another half to comparison (total 10 schools each).

### Intervention

The intervention was based on our previous theory-driven experience over several years. The major theoretical premise underlying the development of these earlier studies was that health promotion must allow participants to take control over the factors that govern their lives, rather than to be driven by pre-determined priorities of outsiders, and that the action for improvement should be based on the participants’ understanding of determinants.

Trained health promotion facilitators visited the intervention schools and delivered the intervention in the form of a series of discussion with the selected students. The discussion was initiated on ‘health’ and ‘wellbeing’ with special emphasis on that of their mothers. Students were introduced to the idea of NCD risk factors (i.e., obesity, sedentary behavior, unhealthy diet, tobacco and alcohol use) and encouraged to discuss these issues in groups and come up with ideas on how to influence them. Facilitators continued to give relevant information at each visit and helped students to work out the determinants of each risk factor—without assistance from their teachers. The facilitator, on subsequent visits, also suggested other determinants that the students had not mentioned. When students thought that the suggested determinants were applicable to their homes, they added such determinants too—for example, scenes from popular television series that promote unhealthy habits. Students were guided to decide how they could measure the selected risk factors and determinants, as applicable to their own homes. They next started to encourage family members to address those determinants they identified in the form of raising questions with their parents and sharing ideas about health and its determinants. Parents were engaged, rather than led, to take action, which was an outcome of shared shift in understanding and interest to do something among members of the household.

Children in all groups documented the changes in health status and lifestyle of their parents—such as weight, participation in exercise, time on sedentary behaviors (especially watching TV), and dietary practice. Additional factors chosen by some groups were also addressed and documented by groups that had added extra ones, such as subjective ‘happiness’ and money saved. Mothers were continually involved in keeping track of their own progress through the measurements that the children made periodically. Children were trained to provide feedback to their mothers.

When students became energized by creating changes within their own home, they were encouraged to reach neighborhood homes too, to explore the potential of spreading the results to the community, beyond the household. Some community level activities initiated by the students were evident, though not formally assessed. In most instances, parents (mostly mothers) also made an effort to involve neighboring households. Apart from individual level activities, the involvement of other families led to shared participation in games and exercise. The involvement in these shared activities and discussions about health led to creating groups of families which took up other wider issues—such as common facilities for play and games, time spent on watching television, and the like.

Intervention continued for 12 months. Initial visits by the facilitators to the schools for interventional activities were once a fortnight. The frequency of facilitator visits to the schools was reduced to once in three weeks or four after about five months. The original expectation was to deliver the training after school hours; however, the interest and cooperation that we received from the intervention schools allowed us to do most of the facilitations during school time. The schools made adjustments to teaching slots and free periods to allow our facilitators access to the intervention students by prior arrangement. As a consequence, students’ participation rate into the program was high. The duration of a typical class was approximately 45 min. In the final phases, facilitator visits to communities were mostly at the invitation of the students or families. Children of schools in the control group did not receive any intervention.

Several measures were adopted to ensure the uniformity of the general principles for generating the required process in the intervention. The interventions in all schools were delivered by facilitators who were trained and observed in role play and real life. The time spent by facilitators at each school was documented. Regular visits were initially predetermined, but were later subject to a demand-driven frequency. The eventual variation in time provided to different schools indicated that the variation was not wide.

### Primary and secondary outcomes

Primary outcomes were changes of the following measurements between baseline and 12th month follow-up surveys: (1) weight and BMI, (2) self-reported level of total physical activity and the number of steps per day based on the reading of pedometer, and (3) self-reported consumption of green leafy vegetables, other vegetables, citrus fruits, yellow fruits, other fruits, whole grain product, pulse as main dish, deep fried foods and sugar-sweetened beverages. Secondary outcome was self-reported household purchase per month of cooking oil, sugar, biscuits and ice cream. Although primary and secondary outcomes were not specified in the study protocol, the classification of the above variables were decided, prior to analysis, according to the objective of the study.

### Outcome measurements

For the assessment of outcomes and other related factors in both intervention and control group of mothers of the selected school children, baseline survey was conducted prior to intervention and follow-up survey was conducted immediately after the completion of intervention (12 month after the initiation of intervention). These surveys were done by a team of three trained staff, who were not involved in the intervention, according to a standard protocol. Body weight and height were measured in a light clothing without shoes. Weight was measured using a digital weighing scale (Seca, Germany) and recorded to the nearest 0.2 kg. Height was measured using a portable stadiometer (Seca, Germany) and recorded to the nearest 0.1 cm. BMI was calculated as weight (kg) divided by the square of height (m). Physical activity was measured by using a Sinhalese version of the International Physical Activity Questionnaire (IPAQ)-long form [13], which has been validated in Sri Lanka [14]. Metabolic equivalents (MET)-minutes per week for total physical activity was calculated according to the scoring system of the IPAQ. We defined “adequate physical activity” as 5359 MET-minutes per week or higher, which corresponded to the upper one-third of total physical activity among the control group. The above cutoff is higher than that of Japanese study among employees (3000 MET-minutes per week) [15] but lower than that of Chinese study (8268 MET-minutes per week) [16], in which nearly half of the participants were recruited from rural area. Mothers wore a pedometer (POWER-WALKER EX-510, YAMAX, Tokyo) for three consecutive days prior to each survey. The survey staff collected the pedometer, read the number of recorded steps for each day, and averaged these figures. If step counts were not recorded on any of the three days or extremely low or high (<500 or >100,000 steps), such data were discarded and the remaining ones were averaged. Diet was assessed using a 27-item self-administered food frequency questionnaire that was developed and validated in Sri Lanka [17], which was supplemented with additional questions on the consumption of other foods and beverages as well as household purchase of cooking oil, sugar, biscuits and ice cream.

### Statistical analysis

We identified some extreme figures in the response to physical activity questions. We thus set the maximum value for each sub-domain of physical activity (16,380 MET-minutes per week, 9198 MET-minutes per week, 18,900 MET-minutes per week and 15,918 MET-minutes per week for work, active transportation, domestic and garden and leisure-time activity, respectively) and applied them to such implausible answers. Participants were analyzed based on the group to which they were originally allocated. Main analyses were done among those who completed both baseline and follow-up surveys (completer analysis). We repeated the analyses among all participants including non-completers, for whom missing data of the follow-up survey were replaced with baseline data (last observation carried forward [LOCF] analysis). Demographic and outcome variables were expressed as mean (standard deviation [SD]) or median (interquartile range [IQR]) for continuous data and the number (percentage) of participants for categorical data. The mean (SD) or median (IQR) of the change between baseline and follow-up surveys was presented for continuous outcome variables. We used multilevel models to account for the clustering of mothers within schools. Multi-level multivariable linear regression was used to assess the impact of intervention on continuously measured outcomes, and multi-level multivariable logistic regression was used for binary outcomes, with adjustment for ethnicity and each outcome value at baseline. Continuous variables with skewed distribution (step counts, household purchase of cooking oil, sugar, biscuits and ice cream) were log-transformed to approximate normality before the analyses. Mean with its 95 % confidence interval (CI) and odds ratio with its 95 % CI were calculated for not-skewed continuous and binary variables of outcome, respectively. As regards total physical activity, we performed a sensitivity analysis after excluding those with extreme data. A significant level of 5 % (two sided) was used for all analyses. All statistical analyses were performed using SAS version 9.3 for Windows (SAS Institute, Inc., Cary, UC, USA).

## Results

**Fig. 1:**
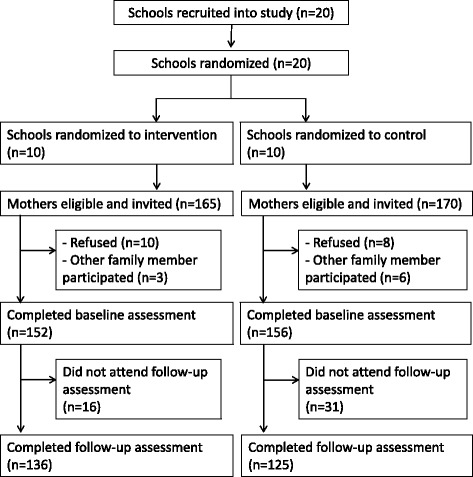
Study flow diagram

Figure 1 shows a study flow diagram showing the number of clusters and participants at each phase of the trial, according to the CONSORT statement. A total of 20 schools were randomized to either the intervention (10 schools) or the control (10 schools), and 335 eligible mothers (165 intervention and 170 control) were invited to the baseline survey. Eighteen mothers refused to participate in the survey (10 intervention and 8 control). Furthermore, we excluded 9 participants (3 intervention and 6 control) because other family member participated in the baseline survey. Of 308 mothers (152 intervention and 156 control) who completed the baseline survey, 47 mothers (16 intervention and 31 control) did not attend the follow-up survey at 12 months, leaving 261 mothers (136 intervention and 125 control) for completer analyses.

Compared with participants who attended the follow-up survey (completers), those who did not attended it (non-completers) were younger (mean age, 30. 2 years vs. 38.0 years) and more likely to be a housewife (83.0 % vs. 58.6 %). The two groups were similar in terms of other demographic factors, body weight, diet and physical activity (Additional file 1: Table S1).

Of completers of the follow-up survey, the intervention and control groups were similar in terms of baseline characteristics except physical activity (Table 1). Mean (SD) age was 37.5 (5.6) years in the intervention group and 38.5 (5.9) years in the control group. In both groups, more than 90 % were Sinhalese and Buddhist, more than half completed junior high school, nearly 60 % were housewives, and nearly 70 % had a household income less than 40,000 Rp per month. Mean (SD) weight was 56.4 (10.8) kg and 55.7 (9.2) kg and mean BMI (SD) was 24.4 (4.6) kg/m^2^ and 24.1 (3.9) kg/m^2^ for the intervention and control groups, respectively. The proportion of overweight and obese was 29.4 and 10.3 %, respectively, in the intervention group and 30.4 and 7.2 %, respectively, in control group. The proportion of having adequate physical activity was 46.6 % in the intervention group and 33.6 % in the control group at baseline. The median (IQR) number of steps per day was 7163 (4930–11179) and 6492 (4426–8848) for the intervention and control groups, respectively.

**Table 1:** Baseline characteristics of study participants

	Intervention group	Control group
No. of participants^a^	136	125
Demographic variable		
Age in years, mean (SD)	37.5 (5.6)	38.5 (5.9)
Ethnicity		
Sinhalese	128 (94.1)	124 (99.2)
Tamil	7 (5.2)	1 (0.8)
Muslim	1 (0.7)	0 (0.0)
Religion		
Buddhism	125 (91.9)	121 (96.8)
Hindu	6 (4.4)	1 (0.8)
Roman Catholic/Christian	4 (2.9)	3 (2.4)
Islam	1 (0.7)	0 (0.0)
Education attainment		
Primary level (Grade 1–5)	43 (31.6)	28 (22.4)
Junior high school	72 (52.9)	73 (58.4)
High school or higher	21 (15.4)	24 (19.2)
Household income		
≤ 40,000 Rp/month	100 (73.5)	84 (67.2)
40,001–60,000 Rp/month	24 (17.7)	28 (22.4)
≥ 60,001 Rp/month	12 (8.8)	13 (10.4)
Occupation		
Housewife	79 (58.1)	74 (59.2)
Employed	57 (41.9)	51 (40.8)
History of diabetes	14 (10.3)	7 (5.6)
History of hypertension	8 (5.9)	3 (2.4)
History of dyslipidemia	7 (5.2)	3 (2.4)
Outcome variable		
Weight in kg, mean (SD)	56.4 (10.8)	55.7 (9.2)
Body mass index in kg/m^2^, mean (SD)	24.4 (4.6)	24.1 (3.9)
Obesity level		
Lean (BMI <18.5 kg/m^2^)	10 (7.4)	10 (8.0)
Normal (BMI 18.5–24.9 kg/m^2^)	72 (52.9)	68 (54.4)
Overweight (BMI 25.0–29.9 kg/m^2^)	40 (29.4)	38 (30.4)
Obese (BMI ≥30 kg/m^2^)	14 (10.3)	9 (7.2)
Dietary intake (>4 times/week)		
Green leafy vegetables	98 (72.1)	95 (76.0)
Other vegetables	115 (84.6)	106 (84.8)
Citrus fruits	40 (29.4)	37 (29.6)
Yellow fruits	50 (36.8)	45 (36.0)
Other fruits	86 (63.2)	74 (59.2)
Whole grain product	80 (58.8)	74 (59.2)
Pulse as main dish	10 (7.4)	12 (9.6)
Deep fried foods	10 (7.4)	9 (7.2)
Sugar-sweetened beverages	11 (8.1)	16 (12.8)
Household purchase (/month)		
Oil (bottles, median, IQR)	2 (1, 3)	2 (1, 3)
Sugar (kg, median, IQR)	4 (3, 6)	4 (2, 6)
Biscuits (packets, median, IQR)	5.5 (4, 16)	6 (3, 16)
Ice cream (litters, median, IQR)	1 (0, 4)	1 (1, 3)
Physical activity		
Total (MET-min/week, median, IQR)	4812 (1641, 9930)	3360 (1343, 7089)
Adequate (≥5359 MET-min/week)	61 (46.6)	42 (33.6)
No. of daily steps (median, IQR)	7163 (4930, 11179)	6492 (4426, 8848)

Data are numbers (percentages) unless otherwise indicated

*BMI* body mass index, *IQR* inter-quartile range, *MET* metabolic equivalent, *SD* standard deviation

^a^No. of intervention and control groups were 131 and 125, respectively for physical activity and 122 and 100, respectively for steps

Table 2 shows the differences in the outcome variables at the 12 month follow-up survey between intervention and control groups. The intervention group had a significantly lower mean of weight and BMI than did the control group (*P* <0.0001); the mean (95 % CI) effect comparing the intervention with the control group was −2.49 (−3.38 to −1.60) kg for weight and −0.99 (−1.40 to −0.58) kg/m^2^ for BMI. The intervention group had a significantly higher odds of engaging in adequate physical activity than the control group; multivariable-adjusted odds ratio for intervention versus control was 3.25 (95 % CI 1.87–5.62), which did not greatly change after excluding 29 participants with extremely high physical activity (odds ratio 3.56, 95 % CI 1.98–6.38). Of physical activity sub-domains, only leisure-time activity showed a significant difference between the groups (*P* <0.0001, data not shown in table). The intervention group showed a significantly greater increase in the number of daily steps than the control group (*P* <0.0001). As for diet, there was no significant difference in individual-level food consumption between the two groups after the intervention, but the intervention group showed a significant decrease in household-level purchase of biscuits and ice cream than the control group (*P* <0.0001 and *P* = 0.03, respectively). Results were materially unchanged after additional adjustment for each or a set of demographic factors (data now shown). In LOCF analysis including both completers and non-completers, similar results were obtained (Additional file 2: Table S2).

**Table 2:** Effect of intervention on primary and secondary outcomes at 12th month follow-up: completer analysis

	Intervention group			Control group			Between-group difference at follow-up	
Outcomes (primary/secondary)	*n*	Mean (SD), median (IQR) or number (percentage)	Mean (SD) or median (IQR) of change from baseline	*n*	Mean (SD), median (IQR) or number (percentage)	Mean (SD) or median (IQR) of change from baseline	Difference in means or odds ratio (95 % confidence interval)^a^	*P* value
Continuous outcomes (primary)								
Weight (kg)	136	54.8 (10.0)	−1.7 (2.8)	125	56.6 (9.6)	0.9 (2.7)	−2.49 (−3.38, −1.60)	<0.0001
BMI (kg/m^2^)	136	23.7 (4.4)	−0.7 (1.2)	125	24.4 (4.1)	0.3 (1.2)	−0.99 (−1.40, −0.58)	<0.0001
No. of daily steps	122	8026 (5750, 9919)	348 (−3340, 3238)	100	4999 (3446, 7103)	−1588 (−3334, 915)	-	<0.0001
Continuous outcomes (secondary)								
*Household purchase (per month)*								
Cooking oil (bottles)	134	2 (1, 4)	0 (−1, 1)	125	2 (1, 4)	0 (−1, 2)	-	0.22
Sugar (kg)	136	3 (2, 4)	−1 (−3, 0)	125	4 (2, 6)	0 (−1, 2)	-	0.86
Biscuits (packets)	136	4 (1, 10)	−3 (−12, 1)	125	8 (5, 10)	0 (−11, 6)	-	<0.0001
Ice cream (litters)	136	1 (0, 2)	0 (−2, 0)	125	3 (1, 5)	1 (−1, 3)	-	0.03
Binary outcomes (primary)								
*Physical activity*								
Adequate (≥5359 MET-min/week)	133	67 (50.4)		125	29 (23.2)		3.25 (1.87, 5.62)	<0.0001
*Dietary intake (>4 days/week)*								
Green leafy vegetables	136	101 (74.3)		125	84 (67.2)		1.47 (0.54, 3.98)	0.45
Other vegetables	136	96 (70.6)		125	84 (67.2)		1.30 (0.53, 3.18)	0.57
Citrus fruits	136	47 (34.6)		125	41 (32.8)		1.09 (0.62, 1.94)	0.77
Yellow fruits	136	58 (42.7)		125	46 (36.8)		1.38 (0.74, 2.56)	0.31
Other fruits	136	76 (55.9)		125	48 (38.4)		2.10 (0.94, 4.68)	0.07
Whole grain product	136	42 (30.9)		125	50 (40.0)		0.72 (0.43, 1.21)	0.21
Pulse as main dish	136	7 (5.2)		125	11 (8.8)		0.62 (0.21, 1.86)	0.39
Deep fried foods	136	5 (3.7)		125	5 (4.0)		0.81 (0.22, 3.04)	0.75
Sugar-sweetened beverages	136	10 (7.4)		125	11 (8.8)		0.73 (0.29, 1.84)	0.50

*BMI* body mass index, *IQR* inter-quartile range, *MET* metabolic equivalent, *SD* standard deviation

^a^Multilevel linear regression for continuous outcomes and multilevel logistic regression for binary outcomes, with school as the cluster variable and adjustment for ethnicity and each outcome value at baseline

## Discussion

In this randomized controlled trial among Sri Lankan mothers with a school-aged child, the intervention group of mothers whose child was educated to be able to act as a change agent in their family showed a statistically significant reduction in weight and increase in physical activity compared with the control group of mothers whose child did not receive any intervention. They also showed a greater reduction of household purchase of biscuits and ice cream. The present study is unique in that intervention was directed at children, who were trained to identify risk factors of NCDs in their home and persuade their mothers to improve lifestyles. This is among few controlled trials that examined the effectiveness of school-based intervention for NCD risk reduction of parents.

In a randomized controlled trial in Brazil [9], Fornari et al. showed that an educational program in cardiovascular disease prevention for school children, aged 6–10 years old, improved the Framingham cardiovascular risk of their parents. In a school-based randomized trial in China [10], an educational program for children was shown to decrease salt intake and blood pressure among adults of their family. An observational study in the U.S. on cancer screening showed that daughters were able to successfully recall and deliver a cancer appeal to their mother [18], and a one-arm intervention study in Japan showed that a stroke education program for elementary school children improved the knowledge of their parents about signs and risk factors of stroke [19]. Additionally, Evans et al. reported that children’s participation in a school-based program to improve the asthma self-management skills of their parents [20]. The present findings, together with previous ones, suggest that “child-to-parent” approach is feasible and can contribute to the primary and secondary prevention of NCDs among family members. The present study adds to the evidence of school-based, child-initiated approach to decrease NCD risk of their mothers in a developing country, where the resource of NCD prevention is limited.

In Sri Lanka, like other developing countries, there remains a social norm about the role of mothers, especially in rural area; mother should stay at home performing domestic duties [21]. Such a norm, together with an expansion of motorization, might have driven Sri Lankan mothers to be inactive on leisure and commuting. On the other hand, energy-dense foods and sugar-sweetened beverages are easily available at modest cost. Exposure to such psychosocial and physical environmental factors together may increase the risk of obesity among Sri Lankan women, especially when the knowledge about the prevention of NCDs is lacking. The present intervention program was designed to address these issues through the actions of children, who were empowered to identify NCD risk factors at their home, find solutions to change the factors identified, and keep records of the changes as they occurred.

Current evidence is inconclusive on whether community-based intervention can increase physical activity among women [22], suggesting a need for innovation in physical activity intervention for women. In the present study, mothers of the intervention arm showed an increase of physical activity, especially during leisure-time. This result is compatible with the records kept by students, who documented mothers’ activity during the course of intervention as well as observation by facilitators. For instance, some mothers began exercise programs on voluntary basis, and later invited other family members including children. Interested mothers came to the school playground to exercise, on invitation of their children, during the late afternoons and weekends. Some families started their own play activities or games at home—such as badminton, playing ‘catch’ and walking. Enthusiastic mothers got their neighbors involved into their groups. Some mothers started enjoying cultivation, gardening and other outdoor home activities, instead of being sedentary at home. Given an important role of social support for the improvement of mothers’ lifestyle [23], physical activity together with children and community members might have helped mothers initiate and continue their activity.

With regard to diet, we found limited evidence supporting favorable influence of the present intervention on dietary habit of mothers, with only a suggestion of greater increase of consumption of “other fruits” compared with control group. This finding is, however, potentially important given that in Sri Lanka fruit consumption has been shown to be below the recommended level [24], although fruits are available across seasons. There was also a significant reduction of household purchase of biscuits and ice cream among mothers in the intervention group. In keeping with suggestions from facilitators, some mothers collected money that was not spent as before on sugar, cooking oil, flour and cigarettes in a special till and invested it to buy needs for community sport activity. The use of such behavioral approach might help mothers to avoid excessive purchase of sweets.

The present study has several strengths, including randomized controlled design, child-initiated health promotion addressing multiple determinants of NCD risk in each family, and various social activities providing opportunity of improving lifestyle and creating better psychosocial environment for health promotion in the community. Moreover, the present child-support approach has an advantage over intervention program exclusively for mothers in enhancing family-based activity, making all family members healthier. The study limitations also warrant mention. First, we assessed usual physical activity and diet based on self-report by participants. Although the assessment tools we used have been validated, we cannot exclude a possibility of differential reporting between intervention and control groups. Specifically, mothers in the intervention group might be more likely to give a favorable answer to these questions due to their expected change of behaviors, compared with those in the control group. Nevertheless, we also observed greater improvement in the intervention group for outcomes that were measured objectively (i.e., step count and body weight). As regards diet, the food frequency questionnaire we used may not be sensitive enough to capture a modest change of food consumption. Additionally, we were unable to assess the amount of sugar and oil consumption at individual level. Second, we instructed participants to wear a pedometer throughout the day, but we did not collect information on how long participants wore the pedometer each day. We thus cannot perform sensitivity analysis among those who wore the pedometer for a sufficient length of time. Third, we assessed the effect of intervention in comparison with no intervention. It is thus unclear whether child support would give additional benefit for mothers compared with an intervention on mothers only (without child support). Fourth, the present study has a 12-month intervention and observation period, and the long-term effect of this program is unclear. Finally, as the present study was performed among mothers of school-aged children of grade 8 (aged around 13 years) in semi-urban area of Colombo, it remains unclear whether this type of program is feasible and can work similarly if the intervention is provided among mothers with children of other ages or in urban or rural settings.

## Conclusions

In this 12-month randomized controlled trial in Sri Lankan mothers with a school-aged child, the intervention to motivate students to change their mothers’ lifestyle was effective in increasing physical activity and decreasing weight of mothers as well as in decreasing the amount of household purchase of biscuits and ice cream. The present finding provides a scientific basis for a “child-to-parent” approach, where students are trained to identify risk factors of NCDs in their respective homes and seek solutions for improvement using local resources, as a strategy for the prevention of NCDs in developing countries. Future study should explore whether such child-initiated or child-involved approach can improve behaviors and health status of other family members and neighborhoods as well as of students themselves.

## Additional files

Additional file 1: Table S1. Baseline characteristics of completers and non-completers. (DOC 59 kb) Additional file 2: Table S2. Effect of intervention on primary and secondary outcomes at 12th month follow-up: LOCF analysis. (DOC 42 kb)

## Abbreviations

BMI: body mass index
CI: confidence interval
IPAQ: international physical activity questionnaire
LOCF: last observation carried forward
MET: metabolic equivalents
NCD: noncommunicable disease
SD: standard deviation
