# Supplementary material for: School-based intervention to enable school children to act as change agents on weight, physical activity and diet of their mothers: a cluster randomized controlled trial
Source: Int J Behav Nutr Phys Act. 2016 Apr 6;13:45. doi: 10.1186/s12966-016-0369-7 (PMC4822262; doi:10.1186/s12966-016-0369-7)
Supplement: Additional file 1: Table S1. — Baseline characteristics of completers and non-completers. (DOC 59 kb) [file 12966_2016_369_MOESM1_ESM.doc]

Additional file 1

Table S1. Baseline characteristics of completers and non-completers

|  | Completers |  | Non-completers |
| --- | --- | --- | --- |
| No. of participants^1^ | 261 |  | 47 |
| Age in years, mean (SD) | 38.0 (5.8) |  | 30.2 (3.6) |
| Ethnicity |  |  |  |
| Sinhalese | 252 (96.6) |  | 47 (100) |
| Others | 9 (3.4) |  | 0 (0) |
| Religion |  |  |  |
| Buddhism | 246 (94.3) |  | 44 (93.6) |
| Others | 15 (5.7) |  | 3 (6.4) |
| Education attainment |  |  |  |
| Primary level (Grade 1-5) | 71 (27.2) |  | 10 (21.3) |
| Junior high school | 145 (55.6) |  | 29 (61.7) |
| High school or higher | 45 (17.2) |  | 8 (17.0) |
| Household income |  |  |  |
| ≤40,000 Rp/month | 184 (70.5) |  | 33 (70.2) |
| 40,001-60,000 Rp/month | 52 (19.9) |  | 9 (19.2) |
| ≥60,001 Rp/month | 25 (9.6) |  | 5 (10.6) |
| Occupation |  |  |  |
| Housewife | 153 (58.6) |  | 39 (83.0) |
| Employed | 108 (41.4) |  | 8 (17.0) |
| History of diabetes | 21 (8.1) |  | 3 (6.4) |
| History of hypertension | 11 (4.2) |  | 4 (8.5) |
| History of dyslipidemia | 10 (3.8) |  | 4 (8.5) |
| Weight in kg, mean (SD) | 56.1 (10.0) |  | 57.6 (10.6) |
| Body mass index in kg/m^2^, mean (SD) | 24.3 (4.3) |  | 25.2 (4.5) |
| Dietary intake (>4 times/week) |  |  |  |
| Green leafy vegetables | 193 (74.0) |  | 31 (66.0) |
| Other vegetables | 221 (84.7) |  | 35 (74.5) |
| Citrus fruits | 77 (29.5) |  | 18 (38.3) |
| Yellow fruits | 95 (36.4) |  | 18 (38.3) |
| Other fruits | 160 (61.3) |  | 31 (66.0) |
| Whole grain product | 154 (59.0) |  | 22 (46.8) |
| Pulse as main dish | 22 (8.4) |  | 4 (8.5) |
| Deep fried foods | 19 (7.3) |  | 4 (8.5) |
| Sugar-sweetened beverages | 27 (10.3) |  | 6 (12.8) |
| Household purchase (/month) |  |  |  |
| Cooking oil (bottles, median, IQR) | 2 (1, 3) |  | 2 (1, 2) |
| Sugar (kg, median, IQR) | 4 (3, 6) |  | 4 (3, 6) |
| Biscuits (packets, median, IQR) | 6 (4, 16) |  | 4 (2, 13) |
| Ice cream (litters, median, IQR) | 1 (1, 3) |  | 2 (0, 4) |
| Physical activity |  |  |  |
| Total (MET-min/week, median, IQR) | 3620 (1440, 8595) |  | 4092 (1620, 9120) |
| Adequate (≥5359 MET-min/week) | 104 (40.3) |  | 20 (40.8) |
| No. of daily steps (median, IQR) | 6734 (4513, 10022) |  | 6357 (4869, 9618) |

^1^ No. of completers and non-completers with baseline measurement were 258 and 49, respectively, for physical activity and 222 and 58, respectively, for steps.

Data are numbers (percentages) unless otherwise indicated.

Abbreviations: IQR, inter-quartile range; MET, metabolic equivalent; SD, standard deviation
