# Supplementary material for: School-based intervention to enable school children to act as change agents on weight, physical activity and diet of their mothers: a cluster randomized controlled trial
Source: Int J Behav Nutr Phys Act. 2016 Apr 6;13:45. doi: 10.1186/s12966-016-0369-7 (PMC4822262; doi:10.1186/s12966-016-0369-7)
Supplement: Additional file 2: Table S2. — Effect of intervention on primary and secondary outcomes at 12th month follow-up: LOCF analysis. (DOC 42 kb) [file 12966_2016_369_MOESM2_ESM.doc]

Additional file 2

Table S2. Effect of intervention on primary and secondary outcomes at 12th month follow-up: LOCF analysis

|  |  | Between-group difference at follow-up | |
| --- | --- | --- | --- |
| Outcomes (primary/secondary) | No. of participants in intervention /control group | Difference in means or odds ratio (95% confidence interval) ^1^ | P value |
| **Continuous outcomes (primary)** |  |  |  |
| Weight (kg) | 152/156 | -2.18 (-2.92, -1.43) | <0.0001 |
| BMI (kg/m^2^) | 152/156 | -0.87 (-1.21, -0.53) | <0.0001 |
| No. of daily steps | 145/135 | － | <0.0001 |
| **Continuous outcomes (secondary)** |  |  |  |
| *Household purchase (per month)* |  |  |  |
| Cooking oil (bottles) | 150/156 | － | 0.35 |
| Sugar (kg) | 152/156 | － | 0.78 |
| Biscuits (packets) | 152/156 | － | <0.0001 |
| Ice cream (litters) | 152/156 | － | 0.07 |
| **Binary outcomes (primary)** |  |  |  |
| *Physical activity* |  |  |  |
| Adequate (≥5359 MET-min/week) | 151/156 | 3.56 (2.12, 5.97) | <0.0001 |
| *Dietary intake (*>*4 days/week)* |  |  |  |
| Green leafy vegetables | 152/156 | 1.30 (0.53, 3.17) | 0.57 |
| Other vegetables | 152/156 | 1.20 (0.58, 2.51) | 0.62 |
| Citrus fruits | 152/156 | 1.04 (0.61, 1.76) | 0.89 |
| Yellow fruits | 152/156 | 1.29 (0.75, 2.23) | 0.35 |
| Other fruits | 152/156 | 1.73 (0.91, 3.30) | 0.09 |
| Whole grain product | 152/156 | 0.69 (0.42, 1.13) | 0.14 |
| Pulse as main dish | 152/156 | 0.64 (0.23, 1.77) | 0.39 |
| Deep fried foods | 152/156 | 1.02 (0.24, 4.40) | 0.98 |
| Sugar-sweetened beverages | 152/156 | 1.28 (0.51, 3.24) | 0.60 |

^1^ Multilevel linear regression for continuous outcomes and multilevel logistic regression for binary outcomes, with school as the cluster variable and adjustment for ethnicity and each outcome value at baseline.

Abbreviations: BMI, body mass index; LOCF, last observation carried forward; MET, metabolic equivalent
